# Regulation of Glutamatergic Activity via Bidirectional Activation of Two Select Receptors as a Novel Approach in Antipsychotic Drug Discovery

**DOI:** 10.3390/ijms21228811

**Published:** 2020-11-20

**Authors:** Paulina Cieślik, Joanna M. Wierońska

**Affiliations:** Department of Neurobiology, Maj Institute of Pharmacology Polish Academy of Sciences, 12 Smętna Street, 31-343 Krakow, Poland; cieslik@if-pan.krakow.pl

**Keywords:** schizophrenia, metabotropic glutamate receptors, muscarinic receptors, GABA_B_ receptor

## Abstract

Schizophrenia is a mental disorder that affects approximately 1–2% of the population and develops in early adulthood. The disease is characterized by positive, negative, and cognitive symptoms. A large percentage of patients with schizophrenia have a treatment-resistant disease, and the risk of developing adverse effects is high. Many researchers have attempted to introduce new antipsychotic drugs to the clinic, but most of these treatments failed, and the diversity of schizophrenic symptoms is one of the causes of disappointing results. The present review summarizes the results of our latest papers, showing that the simultaneous activation of two receptors with sub-effective doses of their ligands induces similar effects as the highest dose of each compound alone. The treatments were focused on inhibiting the increased glutamate release responsible for schizophrenia arousal, without interacting with dopamine (D_2_) receptors. Ligands activating metabotropic receptors for glutamate, GABA_B_ or muscarinic receptors were used, and the compounds were administered in several different combinations. Some combinations reversed all schizophrenia-related deficits in animal models, but others were active only in select models of schizophrenia symptoms (i.e., cognitive or negative symptoms).

## 1. Introduction

Schizophrenia is one of the most complicated mental disorders, and it is characterized by different symptoms that may enrich or disrupt normal behavior. Particular symptoms are not equally manifested in patients, and at least four groups of patients with schizophrenia have been described. However, diagnostic manuals (DSM-V and ICD-11) have recently abandoned the use of schizophrenia subtypes, as they are not stable over time, have low diagnostic value, and substantially reduce the heterogeneity of schizophrenia [1,2]. Separate diseases characterized by schizophrenia-like symptoms have also been specified. The manifestation, intensity, and occurrence of particular symptoms differ between groups (Table 1).

A large percentage of patients with schizophrenia suffer from cognitive impairments that substantially influence daily functioning. Patients with severe cases of schizophrenia or individuals with the predominant presentation of negative and cognitive symptoms are generally treatment-resistant. Other patients with schizophrenia, who respond relatively well to antipsychotic medications, develop adverse effects that lead to discontinuation of the treatment. These factors make living with schizophrenia difficult or impossible. In contrast to other mental diseases, such as depression or anxiety, the effectiveness of psychotherapy as an add-on treatment to antipsychotic medication is very limited [4,5].

Dopamine (D_2_) receptor blockade is the basic mechanism of action of currently used neuroleptic drugs. This receptor is responsible for drug efficacy and the development of adverse effects [6,7]. In contrast to typical neuroleptics with affinity for dopaminergic receptors only, the mechanisms of action of newer generations of drugs, also called atypical neuroleptics, involve a dopamine-based mechanism of action and antagonism or agonism towards serotonergic, adrenergic or histaminergic components [8]. However, atypical antipsychotics remain a heterogeneous group that exhibits different binding profiles, with risperidone being the least and clozapine the most atypical drug [9,10,11]. Diverse targets render atypical drugs slightly more effective and better tolerated [12], but the problem of drug resistance in patients with severe cases of schizophrenia, and the risk of the occurrence of adverse effects, remain relatively high.

The search for new treatment strategies for schizophrenia began years ago, but no spectacular achievements have been reported. This lack of success may be partially due to the ambiguous, unspecified, and complex causes of schizophrenia arousal. The specific changes responsible for schizophrenia development that contribute to the manifestation of particular symptoms have not been fully determined. For many years, the dopaminergic theory of schizophrenia dominated the field and indicated increased dopaminergic neurotransmission as the main factor responsible for the pathophysiology of the disease [8]. The theory was proposed based on observations that dopaminergic antagonists reversed the psychotic symptoms of schizophrenia [13,14,15]. The lack of effectiveness of dopamine-based drugs towards negative and cognitive symptoms of schizophrenia caused doubts regarding the theory and indicates obvious shortcomings of the hypothesis and limits of the treatment. Further research indicated that changes in dopaminergic neurotransmission were not necessarily crucial in schizophrenia arousal. At least two groups of patients were distinguished that differed in their responsiveness to treatment [16]. These groups were normodopaminergic and hyperdopaminergic subpopulations of patients. The latter group had a better response to neuroleptic medications [16]. Genetic predispositions were also indicated as important in successful treatment [17,18,19].

The observations that NMDA receptor antagonists, such as PCP, ketamine, or dizocilpine (MK-801), induced the full spectrum of schizophrenia symptoms prompted the development of the glutamatergic hypothesis of schizophrenia [20,21,22,23]. One of the first papers describing its more important relevance was released in 1987 by Javitt et al., who reviewed studies showing the induction of negative symptoms of schizophrenia in healthy subjects and animals after PCP administration and proposed a novel hypothesis of schizophrenia [24]. Other studies also presented this hypothesis and suggested that preferential hypofunction of NMDA receptors expressed on GABAergic postsynaptic sites led to a decrease in the sensitivity of these neurons to the stimulatory effect of glutamate [25,26]. Consequently, the synthesis and release of GABA becomes impaired, and the subsequent inhibitory control over glutamatergic neurons is lost. The resulting increase in glutamate release is the proposed primary cause of schizophrenia development and results from the hypofunction of NMDA receptors at critical sites in local circuits that modulate the function of a particular brain region or control projections from one region to another (e.g., hippocampal–cortical or thalamocortical projections) [25,26]. This increased glutamate efflux under specific conditions or individual predisposition results in subsequent changes in other neurotransmitters, e.g., dopamine [15].

The formulation of this theory provided new possibilities in the search for treatment strategies based on the reduction of enhanced glutamatergic transmission. Naturally occurring full or partial agonists at the glycine modulatory site of the NMDA receptor, such as glycine, d-serine, and d-cycloserine, and a glycine transporter inhibitor with low affinity, sarcosine, were investigated in add-on studies to ongoing antipsychotic treatment and primarily focused on persistent negative symptoms [27]. Improvements in negative symptoms, sometimes with improvements in cognitive and positive symptoms, were noted [28,29,30,31,32,33], although subsequent meta-analyses did not confirm these results [27,34]. However, the activation of NMDA-dependent pathways with dopaminergic system inhibition and the activation/inhibition of accidental receptors confound the therapeutic effect and increase the risk of adverse effects.

The discovery of metabotropic glutamate (mGlu) receptors in 1989 showed the possibility of regulating glutamatergic neurotransmission without directly targeting NMDA ion channels.

Extensive research on the therapeutic potency of mGlu receptors and their distribution within the CNS is summarized in a vast number of review papers. A PubMed search of “schizophrenia” and “metabotropic glutamate receptors” retrieved more than 100 review papers. The most important reviews are shown in Table 2.

Despite the massive effort and financial resources invested to develop and introduce antipsychotic drugs with a mechanism of action based on the stimulation of mGlu receptors, a confirmed successful clinical trial has not been reported. After the controversial data published by Kinon et al. [56] and Patil et al. [57], clinical studies on a new generation of antipsychotics targeting mGlu receptor ligands were strongly limited but not completely discontinued. Therefore, innovative solutions focused on the inhibition of glutamatergic activity based on mGlu receptor signaling are desired. One possibility is as an add-on therapy based on the concomitant activation of other types of receptors involved in the regulation of the glutamatergic network.

## 2. Malfunction of Receptors in Patients with Schizophrenia

The causes of the pathophysiology of the disease and the subsequent changes that develop must be recognized and are fundamental to determining and introducing safe and effective treatments. Disrupted synaptic organization or impairments in receptor expression and function are important factors that may contribute to the success or failure of treatment.

According to some studies, patients with schizophrenia present diminished expression of the RGS4 mRNA [58,59,60,61], which is one of the 30 RGS molecules that function as GTPase activator proteins for Gα subunits. RGS4 is predominantly expressed in the brain [62], and a malfunction in RGS4 molecules translates into dysfunction of the G-protein-mediated signaling of metabotropic glutamate [63], GABAergic [64] and muscarinic acetylcholine receptors [65]. Available data and postmortem studies revealed robust changes in the expression of these receptors in patients with schizophrenia (Table 3A–C).

Most studies indicated decreased expression of mGlu_2_ receptors in the hippocampus of patients with schizophrenia, but increased expression in the cortex was also observed (Table 5C). Similarly, GABA_B_, M_1_, and M_4_ receptors were downregulated in most studies, and a few studies reported no changes (Table 3A,B). No changes in the expression of mGlu_4_ or the mGlu_5_ receptor were observed in postmortem studies (Table 3C). The functionality or excitability of these receptors is not known in patients with schizophrenia.

Statistical comparisons revealed robust changes and global trends in the population. Notably, individual features related to receptor expression and functionality made individual patients more susceptible to the development of specific symptoms of the disease and determined the responsiveness to treatment. Although the general trends of the population indicate the most plausible effective solutions, these solutions may fail in individual patients. Many different hypotheses have been proposed to explain why some individuals respond better than others to treatment, but the exact mechanisms of these discrepancies are not known [66,67]. However, differences in the expression and functionality of receptors between patients may underlie the differential responses.

The latest few papers published by our group proposed treatment strategies based on the bidirectional activation of select receptors. The strategy was to abolish glutamatergic arousal responsible for schizophrenia pathophysiology via activation of the most relevant pathways.

**Table 3:** Expression of muscarinic (M_1_ and M_4_) (**A**), GABA_B_ (**B**) and metabotropic glutamatergic receptors (mGlu_5_, mGlu_2/3_, mGlu_2_, mGlu_4_, and mGlu_7_) (**C**) in postmortem brain tissues from patients with schizophrenia.

**(A)**				
**Receptor**	**Method**	**Brain Structure**	**Change**	
**M_1_/M_4_**				
	[^3^H] pirenzepine binding	caudate-putamen	decrease	[68]
	[^3^H] pirenzepine binding	hippocampal formation	decrease	[69]
	[^3^H] pirenzepine binding	Brodmann area 9	decrease	[70]
	[^3^H] pirenzepine binding	Brodmann area 40	no change	[70]
	[^3^H] pirenzepine binding	Brodmann area 9	decrease	[71]
	[^3^H] pirenzepine binding	Brodmann area 46	decrease	[71]
	[^3^H] pirenzepine binding	anterior cingulate cortex	decrease	[72]
	[^3^H] pirenzepine binding	superior temporal gyrus	decrease	[73]
	[^3^H] pirenzepine binding	posterior cingulate cortex	decrease	[74]
	[^3^H] pirenzepine binding	hippocampal formation	decrease	[75]
	[^3^H] pirenzepine binding	Brodmann area 6	decrease	[76]
**M_1_**				
	in situ hybridization	caudate-putamen	no change	[77]
	in situ hybridization, Western blot	Brodmann area 9	decrease	[70]
	in situ hybridization, Western blot	Brodmann area 40	decrease no change	[70]
	cDNA	Brodmann area 6	decrease	[78]
	in situ hybridization, Western blot	thalamus	no change	[79]
	in situ hybridization	hippocampal formation	no change	[75]
	immunohistochemistry	Brodmann area 9	decrease	[80]
	immunohistochemistry	Brodmann area 17	decrease	[80]
	immunohistochemistry	thalamus	no change	[80]
	immunohistochemistry	hippocampal formation	no change	[80]
**M_4_**				
	in situ hybridization, Western blot	Brodmann area 9	no change	[70]
	in situ hybridization, Western blot	Brodmann area 40	decrease no change	[70]
	in situ hybridization, Western blot	thalamus	no change	[79]
	in situ hybridization	hippocampal formation	decrease	[75]
**M_2_/M_4_**				
	[^3^H]AF-DX 384	anterior cingulate cortex	no change	[81]
**(B)**				
**Receptor**	**Method**	**Brain Structure**	**Change**	
**GABA_B_**				
	immunohistochemistry	hippocampal formation	decrease (not quantified)	[82]
	immunohistochemistry	entorhinal cortex, inferior temporal cortex	decrease (not quantified)	[83]
	immunohistochemistry, Western blot	Brodmann area 9	decrease (not quantified), decrease (GABA_B1a_)	[84]
	Western blot	lateral cerebellum	decrease	[85]
	Western blot	Brodmann area 9	decrease	[86]
**(C)**				
**Receptor**	**Method**	**Brain Structure**	**Change**	
**mGlu_5_**				
	[^3^H]MPEP binding	Brodmann area 46	no change	[87]
	[^3^H]MPEP binding	Brodmann area 24	no change	[88]
	in situ hybridization	Brodmann area 9	no change	[89]
	in situ hybridization	Brodmann area 10	no change	[89]
	in situ hybridization	Brodmann area 11	increase	[89]
	in situ hybridization	hippocampal formation	no change	[90]
	in situ hybridization	parahippocampal gyrus	no change	[90]
	in situ hybridization	thalamus	no change	[91]
	Western blot	Brodmann area 9	no change	[92]
	Western blot	Brodmann area 11	no change	[92]
	Western blot	Brodmann area 32	no change	[92]
	Western blot	Brodmann area 46	no change	[92]
	Western blot	nucleus accumbens	no change	[92]
	Western blot	caudate nucleus	no change	[92]
	Western blot	putamen	no change	[92]
	Western blot	Brodmann area 10	no change	[93]
	Western blot	lateral cerebellum	decrease (monomer)	[94]
	Western blot	Brodmann area 9	decrease (monomer)	[94]
	Western blot	Brodmann area 46	no change (monomer)	[87]
	Western blot	Brodmann area 46	increase (total and dimer)	[95]
	RT-PCR	Brodmann area 9	no change	[96]
	qRT-PCR	lateral cerebellum	decrease	[94]
	qRT-PCR	Brodmann area 46	no change	[95]
	qPCR	Brodmann area 10	no change	[97]
	qPCR	Brodmann area 46	no change	[97]
**mGlu_2/3_**				
	[^3^H]LY341495 binding	Brodmann area 24	no change	[88]
	[^3^H]LY341495 binding	Brodmann area 17	no change	[98]
	[^3^H]LY341495 binding	Brodmann area 24	no change	[98]
	[^3^H]LY341495 binding	Brodmann area 46	no change	[98]
	[^3^H]LY341495 binding	Brodmann area 46	no change	[99]
	Western blot	Brodmann area 46	no change	[100]
	Western blot	PFC	increase	[92]
**mGlu_2_**				
	in situ hybridization	dentate gyrus	decrease	[101]
	in situ hybridization	CA3	decrease	[101]
	in situ hybridization	CA2	decrease	[101]
	in situ hybridization	subiculum	decrease	[101]
	in situ hybridization	parahipocampal gyrus	decrease	[101]
	in situ hybridization	thalamus	no change	[91]
	in situ hybridization	prefrontal cortex (white matter)	increase	[102]
	in situ hybridization	paranigral nucleus	increase	[102]
	Western blot	prefrontal cortex	no change	[103]
	Western blot	temporal cortex	no change	[103]
	Western blot	motor cortex	no change	[103]
**mGlu_4_**				
	in situ hybridization	thalamus	no change	[91]
	Western blot	Brodmann area 9	no change	[92]
	Western blot	Brodmann area 11	no change	[92]
	Western blot	Brodmann area 32	no change	[92]
	Western blot	Brodmann area 46	no change	[92]
	Western blot	nucleus accumbens	no change	[92]
	Western blot	caudate nucleus	no change	[92]
	Western blot	putamen	no change	[92]
**mGlu_7_**				
	in situ hybridization	thalamus	no change	[91]

## 3. Regulation of Glutamate Release

### 3.1. Glutamatergic Network in the Brain

Glutamate is the most abundant excitatory neurotransmitter in the brain, reaching high concentrations ranging from 5 to 15 µM per gram of tissue [104,105]. The activity of glutamatergic neurons is critical for the proper functioning of the cerebral cortex and the subcortical areas receiving glutamatergic projections.

**Figure 1:**
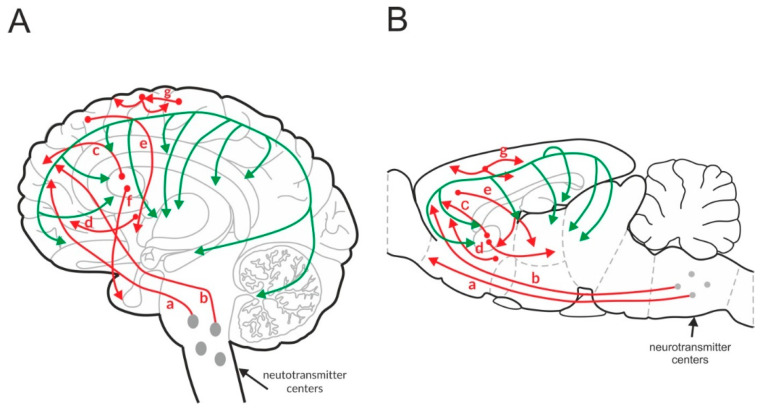
Glutamatergic (**red**) and GABAergic (**green**) pathways in the human (**A**) and rat (**B**) brain. “a” and “b”—cortico-brainstem pathway, “c”—cortico-striatal pathway, “d”—cortico-accumbens pathway, “e”—cortico-thalamic pathway, “f”—thalamo-cortical pathway, and “g”—cortico-cortical pathway.

Glutamatergic neurons are widely distributed across the CNS. At least five key glutamatergic pathways have been identified (Figure 1) [106]. Three pathways descend from the cortex to subcortical structures, such as the brainstem, thalamus, nucleus accumbens, and striatum. One pathway ascends from the thalamus to the cortex. Intracortical loops of glutamatergic interneurons that stabilize the activity of cortical networks have also been identified. Similar loops have been observed in other brain areas, such as the hippocampus.

Based on these connections, glutamate is crucial in the integration of neurotransmission in the brain, including the regulation of monoaminergic nuclei located in the brainstem and cholinergic neurotransmission originating from the pedunculopontine and laterodorsal tegmental nucleus [106,107]. This excitatory system remains under the inhibitory control of GABAergic neurotransmission in a type of homeostatic balance.

GABAergic neurons are spread throughout the brain and form a network that connects with the excitatory system and regulates its functions (Figure 1) [108,109].

A variety of specific mechanisms regulate the release of neurotransmitters. One of the most important mechanisms is the presynaptic regulatory mechanism of receptors expressed on axon terminals, which may involve autoreceptors activated by the transmitters released from the host neuron or heteroreceptors activated by neurotransmitters that are synthesized by other neurons.

The activation or inhibition of receptors localized on dendritic shafts and cell bodies (postsynaptic receptors) triggers an electrical signal by regulating the activity of ion channels. The influx of ions changes the membrane potential of a neuron and results in a signal that is transmitted along the axon to regulate other neurons and the neuronal network.

The most important aspects of the pre- and postsynaptic regulation of glutamatergic networks are summarized below. Attention was placed on receptors that are likely targets for antipsychotic drug discovery.

### 3.2. Presynaptic Regulation of Glutamate Release—Autoreceptors

#### 3.2.1. mGlu_2_ Receptors

The mGlu_2_ receptors are located at a distance from the synaptic cleft [110]. The glutamate potency at mGlu_2_ receptors is high—0.3–20 µM—but mGlu_2_ receptors are exposed to relatively low concentrations of glutamate under physiological conditions [110,111,112]. The receptors are negatively associated with adenyl cyclase activity, and their stimulation results in the inhibition of glutamate release [113].

**Figure 2:**
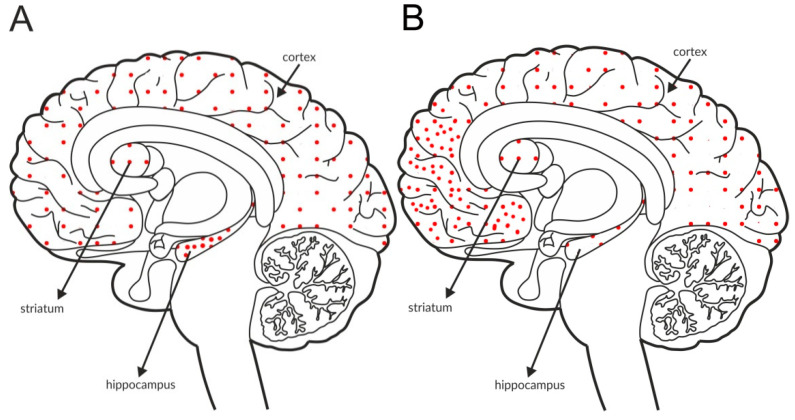
Distribution of mGlu2 receptors in the brains of healthy individuals (**A**) and patients with schizophrenia (**B**). Dotted areas represent receptor expression in select structures. The expression intensity is indicated by the pattern density.

The most intense staining for mGlu_2_ receptors was detected in the neocortex and limbic cortical neurons, predominantly in the hippocampus, as shown in Figure 2A and Table 4A,B. The expression of the receptor at axon terminals was evident, but examples of postsynaptic expression of the receptor on the cell bodies and dendrites of Golgi cells in the cerebellum were also noticed [114].

Some postmortem studies revealed a decrease in the expression of mGlu_2_ receptors in the hippocampus and increased expression in the prefrontal cortex of patients with schizophrenia (Figure 2B, Table 3C).

Ligands activating mGlu_2_ receptors inhibit the release of glutamate and have been extensively investigated as newer antipsychotics in animals and humans. A 2007 article showed the efficacy of a mGlu_2/3_ orthosteric agonist in patients with schizophrenia and provided hope for new treatment solutions [57], as described in the review “Schizophrenia drug says goodbye to dopamine” [115]. Unfortunately, the results from further clinical trials of mGlu_2/3_ orthosteric agonists were far from satisfactory, and work with the compound was ultimately discontinued. However, this decision may have been premature because the ligands displayed excellent activity in preclinical models [51,116] and some clinical studies [117,118].

The conflicting data may result from several factors, such as genetic diversity between humans or a prior history of antipsychotic treatment. Further studies with more homogenous groups of patients and/or without prior medical treatment are needed. Importantly, the poor oral bioavailability of the compounds due to their highly hydrophilic properties was shown to be one of the reasons for their poor efficacy in humans [57,119,120]. One of the solutions to improve the gastrointestinal absorption of compounds is to design prodrugs with better absorption properties. Peptide transporter 1 (PEPT1) regulates the bioavailability of various drugs, including some mGlu_2/3_ agonists; therefore, Eli Lilly designed prodrugs to be absorbed by PEPT1 (LY544344 for LY354740 and LY2140023 for LY404039) [119,121]. The generation of these prodrugs resulted in significantly higher bioavailability of the prototypes [119,122]. However, higher exposure may induce toxicity in patients [123]. An ester-based lipophilic prodrug of another mGlu_2/3_ agonist, MGS0008, was designed to avoid undesirable adverse effects [123]. MGS0274 besylate exhibited a 15-fold improvement in oral bioavailability compared to MGS0008, and its administration to patients was accompanied by fewer toxic effects caused by its unnecessary exposure [120,123,124].

#### 3.2.2. Group III mGlu Receptors

The third group of mGlu receptors consists of the mGlu_4_, mGlu_7_, and mGlu_8_ subtypes. All of these receptors are expressed presynaptically and are negatively associated with adenyl cyclase activity [110]. The potency of glutamate at mGlu_4_ receptors is slightly lower than at mGlu_2_ receptors (3–38 µM), and these receptors are mainly located in the center of the synaptic cleft [110,111], near the site of fusion with synaptic vesicles. Therefore, these receptors are exposed to high glutamate concentrations [112].

**Figure 3:**
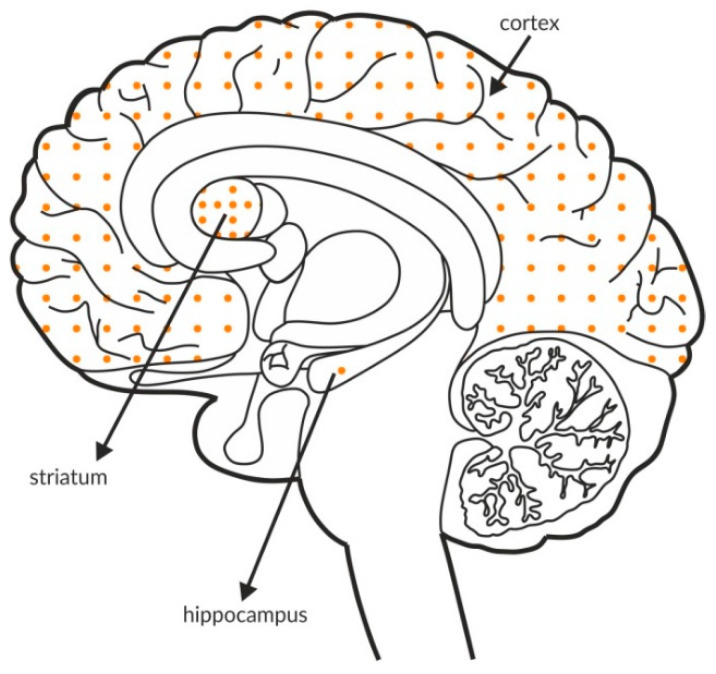
Distribution of mGlu_4_ receptors in the brains of healthy individuals. Dotted areas represent receptor expression in select structures. The expression intensity is indicated by the pattern density.

Similar to mGlu_2_ [125], the mGlu_4_ receptor is expressed predominantly on glutamatergic terminals that oppose other glutamatergic projection neurons [126,127]. At least two splice variants of mGlu_4_ receptors were identified [128], and stimulation of these receptors resulted in antipsychotic efficacy in several studies [51,53]. The receptor is expressed at relatively low levels in the hippocampus and cortex, and the most intense mGlu_4_ labeling is observed in the globus pallidus and cerebellum, as shown Figure 3 and Table 4A,B. Postmortem studies have not shown altered expression of mGlu_4_ receptors in patients with schizophrenia (Table 3C).

The ability of mGlu_2/3_ and mGlu_4_ receptors to inhibit glutamate release in the cortex was confirmed in patch-clamp experiments, in which an orthosteric agonist or positive allosteric modulator (PAM) abolished the frequency (but not the amplitude) of DOI-induced spontaneous EPSCs [129,130,131].

The mGlu_7_ and mGlu_8_ receptors are the least recognized mGlu receptors. Five subtypes of mGlu_7_ [132] and three subtypes of the mGlu_8_ receptor were cloned [133]. Due to the limited number of available ligands activating or inhibiting these receptors, data on their pharmacological activity are scarce. Available publications indicate a lack of efficacy of activation of mGlu_7_ receptors in animal models of schizophrenia [134]. However, the only available mGlu_7_ PAM, AMN082, was only tested in MK-801-induced hyperactivity and DOI-induced head twitches. Therefore, the data are incomplete. In contrast, the efficacy of negative allosteric modulators of the mGlu_7_ receptor was observed in a wide range of tests [135].

The mGlu_7_ receptor is a presynaptic receptor located on glutamatergic axons. However, mGlu_7_-like immunoreactivity was also observed on GAD-expressing neurons in the islands of Calleja or striatum, suggesting that the receptor is also a heteroreceptor on GABAergic neurons [136]. The functional roles of these receptors are not clear because their low affinity for glutamate stimulation at distant synapses by a diluted signal is doubtful.

### 3.3. Presynaptic Regulation of Glutamate Release—Heteroreceptors

Heteroreceptors are activated by neurotransmitters other than those synthesized by the neurons on which the receptors are expressed.

The large number of heteroreceptors involved in the regulation of glutamate release makes a discussion of each type challenging. According to recent data, GABA_B_ and muscarinic M_4_ receptors are of particular importance in the pathophysiology of schizophrenia and antipsychotic drug discovery.

#### 3.3.1. GABA_B_ Receptor

**Figure 4:**
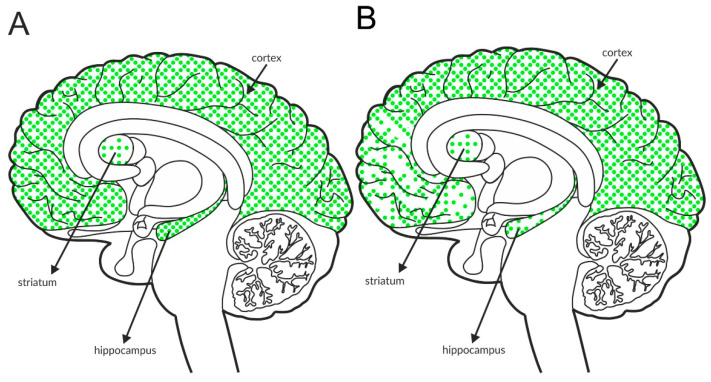
Distribution of GABA_B_ receptors in the brains of healthy individuals (**A**) and patients with schizophrenia (**B**). Dotted areas represent receptor expression in select structures. The expression intensity is indicated by the pattern density.

GABA_B_ receptors, similar to group II and III mGlu receptors, are associated with adenyl cyclase activity and the inhibition of cAMP production. Glutamatergic terminals contain large numbers of this receptor, and its stimulation inhibits glutamate release [137]. Therefore, GABA_B_ receptors, together with mGlu receptors, are one of the most important pathways regulating the release of glutamate. The GABA_B_ receptor is found in all brain areas, and the receptor is expressed at relatively high levels in all brain structures. The labeling of the receptor is higher in the hippocampus and the cortex than in the striatum, with an additional increase in the hippocampus compared with the cortex (Figure 4A and Table 4A,B).

Available postmortem studies revealed decreased expression of GABA_B_ receptors in both the hippocampus and prefrontal cortex of patients with schizophrenia (Figure 4B and Table 3B).

According to preclinical studies, the GABA_B_ receptor is a promising target in antipsychotic drug discovery. The efficacy of PAMs of this receptor has been shown in animal models of positive, negative, and cognitive symptoms [137,138]. Notably, the use of PAMs instead of agonists is recommended because of the lower risk of developing adverse effects, such as myorelaxation or sedation, which may be induced after orthosteric agonist administration [139,140].

#### 3.3.2. Muscarinic M_4_ Receptor

Recently, researchers investigating schizophrenia have focused on muscarinic receptors after the administration of xanomeline was reported to exhibit antipsychotic efficacy in patients with schizophrenia [141]. Xanomeline is a nonselective agonist of muscarinic receptors that preferentially binds to M_1_ and M_4_ receptors [142]. Therefore, this drug also induced adverse effects due to stimulation of peripherally expressed M_2_ and M_3_ receptors [143]. Treatment with selective ligands to activate muscarinic receptor subtypes that are preferentially expressed in the brain, such as M_1_, M_4_, or M_5_, should result in a lower risk of peripherally driven effects. The M_4_ subtype is located at presynaptic sites and may be a heteroreceptor on glutamatergic terminals [144,145].

**Figure 5:**
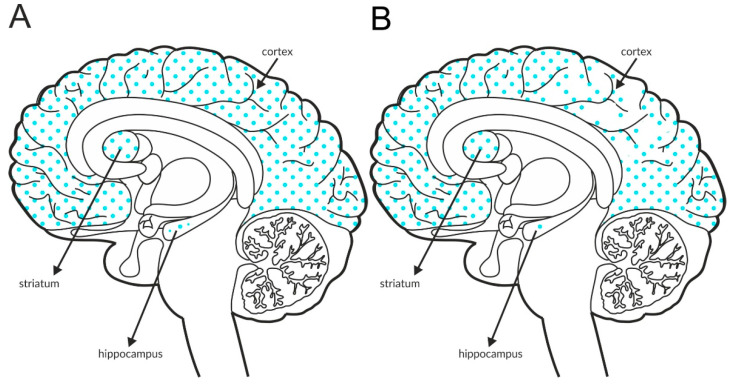
Distribution of M_4_ receptors in the brains of healthy individuals (**A**) and patients with schizophrenia (**B**). Dotted areas represent receptor expression in select structures. The expression intensity is indicated by the pattern density.

The M_4_ receptor is negatively associated with adenyl cyclase activity. It functions as an autoreceptor in the striatum, but it is expressed as a heteroreceptor on glutamatergic axon terminals and regulates glutamate release, predominantly in the cortex and hippocampus [146,147,148,149,150,151]. Patch clamp recordings confirmed its ability to reduce excessive glutamate efflux in the cortex [152]. The expression of the receptor in the structures involved in schizophrenia pathophysiology is shown in Figure 5A and Table 4A,B. Postmortem studies indicate decreased expression of M_4_ receptors in the hippocampus and parietal cortex of patients with schizophrenia (Figure 5B and Table 3A).

### 3.4. Postsynaptic Regulation of Neuronal Circuits in Patients with Schizophrenia

The selection of receptors expressed on cell bodies and dendrites deserves attention in schizophrenia drug development. Their activation changes the neuronal potential and signal transduction along the axon terminal, which may affect distant neurons.

#### 3.4.1. mGlu_5_ Receptor

The mGlu_5_ receptor is a member of the group I metabotropic glutamate receptor family, and it has three splice variants [153]. In contrast to the group II and group III receptors, this subtype interacts with phosphatase C and stimulates inositol production via Gαq signaling.

The mGlu_5_ receptor is expressed near NMDA receptors and is functionally linked via Shank and Homer proteins [154]. Therefore, the stimulation or inhibition of the mGlu_5_ receptor influences NMDA-mediated signaling [155,156,157], indicating that the pharmacological manipulation of this receptor represents a high risk. Fortunately, Conn and coworkers identified that the modulation of NMDA currents was not critical for mGlu_5_ pharmacology and discovered biased, selective potentiators of mGlu_5_ receptors coupled to Gαq-mediated signaling but not mGlu_5_ modulation of NMDAR currents or NMDAR-dependent synaptic plasticity in the rat hippocampus [158]. These ligands bind to sites distinct from the orthosteric (or endogenous) ligand, often with improved subtype selectivity and spatiotemporal control over receptor responses, which constitutes a novel therapeutic approach.

**Figure 6:**
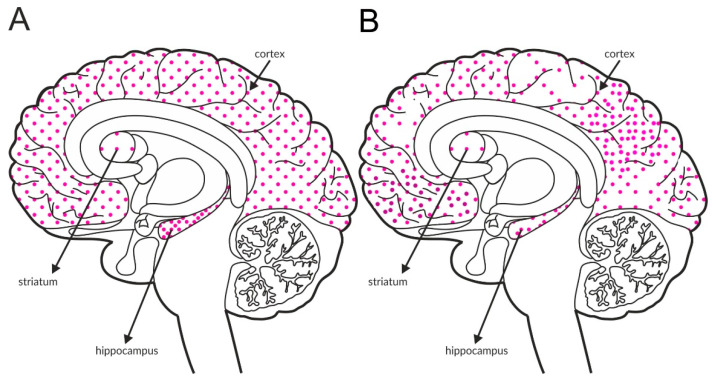
Distribution of mGlu_5_ receptors in the brains of healthy individuals (**A**) and patients with schizophrenia (**B**). Dotted areas represent receptor expression in select structures. The expression intensity is indicated by the pattern density.

The mGlu_5_ receptors generally function as postsynaptic receptors on dendritic spines and shafts, but they were also detected presynaptically on axon terminals in the cortex and hippocampus. Electron microscopy and immunocytochemical studies indicated that these neurons may synthesize GABA [159,160]. The receptor is widely distributed across the brain, including structures that are critical in schizophrenia arousal. The most intense labeling was observed in the hippocampus, followed by the cortex, and the lowest expression was observed in the striatum. A schematic of the distribution of this receptor within these structures in the healthy brain is shown in Figure 6A and Table 4A,B. In postmortem studies, the expression of mGlu_5_ receptors was decreased in the prefrontal cortex and cerebellum (Figure 6B and Table 3C). The data from the frontal cortex are inconclusive, as the expression is increased in some regions and decreased in others.

Stimulation of mGlu_5_ exerted antipsychotic-like activity in a vast range of animal models [51,53].

#### 3.4.2. Muscarinic M_1_ Receptor

**Figure 7:**
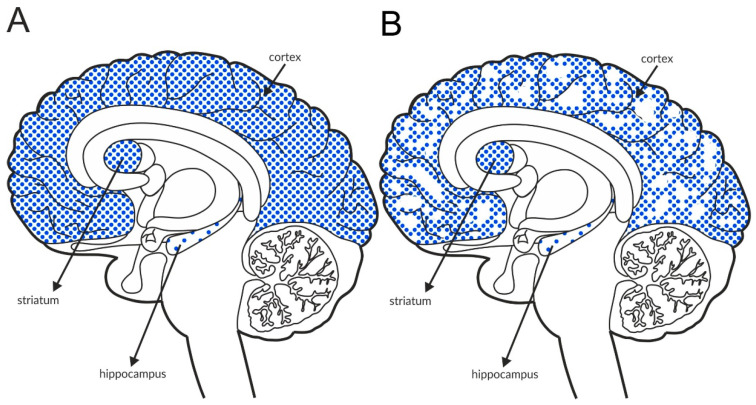
Distribution of M_1_ receptors in the brains of healthy individuals (**A**) and patients with schizophrenia (**B**). Dotted areas represent receptor expression in select structures. The expression intensity is indicated by the pattern density.

The M_1_ receptor is expressed in the cerebral cortex, hippocampus, thalamus, and striatum (Figure 7A and Table 4A,B) [161,162,163,164], and it activates phospholipase C and MAPK in the cerebral cortex in mice [165]. The M_1_ receptor colocalizes with NMDA receptors in hippocampal pyramidal neurons, and the simultaneous activation of the M_1_ and NMDA receptors increases NMDA currents [166]. Deletion of the M_1_ receptor results in a partial impairment of long-term potentiation in the hippocampus [166], which is also reflected in behavior [166,167]. Despite the presence of intact hippocampus-dependent memory, M_1_-/- mice show a deficit in consolidation over time during contextual fear conditioning, as well as impairments in win-shift and social discrimination learning, which suggests a role for the M_1_ receptor in cortex-dependent memory or hippocampal-cortical interaction [166]. M_1_ receptor deletion leads to elevated basal striatal dopamine release and locomotor activity, which is further enhanced by amphetamine challenge [167,168].

The antipsychotic activity of M_1_ receptor ligands has not been extensively tested in preclinical studies. Our studies are some of the first to show activity in animal models of schizophrenia [169]. However, M_1_ ligand activity was observed in models of positive and cognitive, but not negative, symptoms of the disease [169,170].

Postmortem studies revealed decreased expression of M_1_ receptors in various regions of the cerebral cortex in patients with schizophrenia (Figure 7B and Table 3A).

#### 3.4.3. Muscarinic M_5_ Receptor

**Figure 8:**
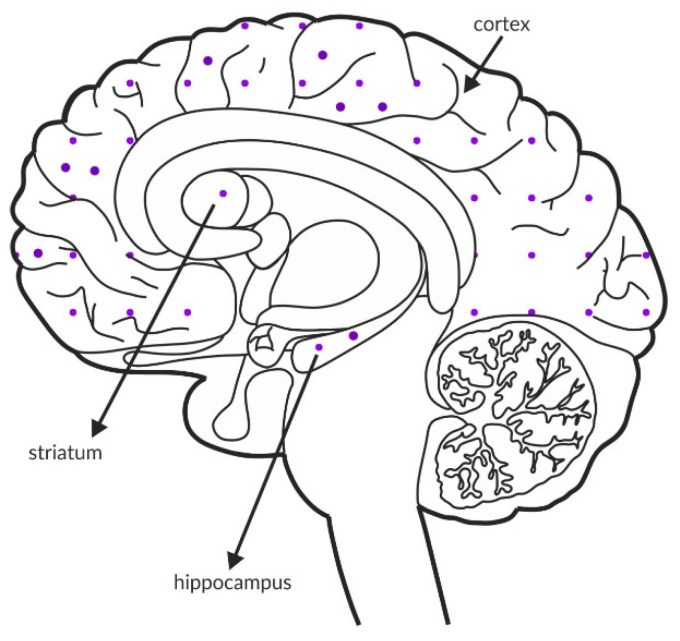
Distribution of M_5_ receptors in the healthy brain. Dotted areas represent receptor expression in select structures. The expression intensity is indicated by the pattern density.

The M_5_ receptor accounts for approximately 2% of all muscarinic receptors in the brain [164], and it is the least studied muscarinic receptor. It is expressed in the hippocampus, hypothalamus, cerebral cortex, striatum, substantia nigra pars compacta and ventral tegmental area (Figure 8 and Table 4A,B) [162,163,171]. It is also found on blood vessels in the brain [172,173]. The location of M_5_ receptors suggests a role in the regulation of dopamine release [174]. These receptors colocalize with D_2_ dopamine receptors in the substantia nigra pars compacta [171]. Due to the lack of selective M_5_ receptor ligands, the first preclinical studies were performed in mice lacking this receptor. The M_5_-/- mice showed no changes in motor coordination or basal locomotor activity, and no significant changes in locomotor activity were observed after amphetamine administration [175]. Deletion of the M_5_ receptor did not affect animal social interactions but weakened sensory motor gating processes [172,176]. M_5_-/- mice also showed a memory impairment in the new object recognition test and the Y maze [172]. The memory impairment may be partially explained by morphological (reduced number of dendritic spines) and physiological (reduced expression of NMDA, AMPA, and kainate receptor subunits, reduced frequency of spontaneous postsynaptic potentials, reduced LTP, and neurotransmitter release disturbances) changes within the hippocampal formation [172]. As shown in our previous studies, a PAM of the M_5_ receptor exerted antipsychotic-like effects on models of positive and cognitive, but not negative, symptoms of schizophrenia [169,170].

#### 3.4.4. Comparative Assessment of Receptor Expression

Table 4A,B summarizes the available data on the expression of particular receptors in rodents and humans. Studies of protein expression were performed using immunohistochemistry, Western blotting and immunoprecipitation, and mRNA expression was investigated using in situ hybridization, PCR, or Northern blotting. All investigated receptors were widely expressed in structures that are important in schizophrenia arousal (e.g., cortex, hippocampus, and striatum).

**Table 4:** The expression of muscarinic (M_1_, M_4_, and M_5_), GABA (GABA_B_), and metabotropic glutamate (mGlu_2_, mGlu_5_, mGlu_4_, mGlu_7_, and mGlu_8_) receptors in the rodent (**A**) or human brain (**B**). Protein expression was determined using immunohistochemistry, Western blotting, and immunoprecipitation. The mRNA levels were assessed using in situ hybridization, PCR, or Northern blotting.

**(A)**				
**Receptor**	**Protein**		**mRNA**	
**M_1_**	cortex (including: mPFC, entorhinal cortex)	[162,170,177,178]	cortex (including piriform cortex, visual cortex)	[171,179,180,181,182,183]
			nucleus accumbens	[171]
	hippocampus	[162,170]	caudate-putamen	[171,179,183]
	caudate-putamen	[162]	basolateral amygdala	[182]
	nucleus accumbens	[162,184]	olfactory tubercule	[179]
	thalamus	[162]	primary olfactory cortex	[182,183]
	amygdala	[162]	hippocampus	[182]
	brainstem	[162]	olfactory nuclei	[182]
	olfactory tubercule	[162]	olfactory bulb	[171,182,183]
**M_4_**	cortex	[162,185]		
	caudate-putamen	[162,184,185]		
	nucleus accumbens	[162]	cortex (including primary olfactory cortex, visual cortex, piriform cortex)	[171,179,180,181,182,183,185]
	thalamus	[162]	nucleus accumbens	[171]
	hippocampus	[185]	caudate-putamen	[171,179,182,183,185]
	substantia nigra	[162]	hippocampus	[182,183,185]
	brainstem	[162]	olfactory tubercule	[171,179,183]
	olfactory tubercule	[162]	olfactory bulb	[182,185]
	olfactory bulb	[185]		
	islands of Calleja	[162]		
**M_5_**	brainstem	[162]	substantia nigra (pc)	[171,186]
			ventral tegmental area	[171,186]
			hippocampus (CA1)	[186]
			ventral subiculum	[186]
**GABA_B_**	cortex	[187,188]	cortex (including piriform cortex)	[189]
	caudate-putamen	[187,188]	hippocampus	[189]
	globus pallidus	[188]	nucleus accumbens	[189]
	nucleus accumbens	[188]	caudate-putamen	[189]
	amygdala	[188]	thalamus	[189]
	hippocampus	[187,188]	hypothalamus	[189]
	thalamus	[187,188]	substantia nigra (pc)	[189]
	hypothalamus	[188]	ventral tegmental area	[189]
	ventral tegmental area	[188]	cerebellum	[189]
	substantia nigra	[188]	pons	[189]
	cerebellum	[187,188]		
	olfactory bulb	[187]	(GABA_B1_)	
	medulla/pons	[187]		
			cortex (including piriform cortex)	[190,191]
	(GABA_B1A_, GABA_B1B_, GABA_B2_)			
			caudate-putamen	[190]
			nucleus accumbens	[190]
			globus pallidus	[190]
			substantia nigra	[190]
			amygdala	[190]
			hippocampus	[190,191]
			hypothalamus	[190]
			thalamus	[190,191]
			cerebellum	[190,191]
			ventral tegmental area	[190]
			pons	[190]
			(GABA_B2_)	
			cortex (including piriform cortex, frontal cortex, occipital cortex, retrosplenial cortex,	[192]
			temporal cortex)	
			hippocampus	[192]
			thalamus	[192]
			hypothalamus	[192]
			striatum	[192]
			nucleus accumbens	[192]
			substantia nigra	[192]
			amygdala,	[192]
			cerebellum	[192]
			(GABA_B1A_, GABA_B2_)	
**mGlu_5_**	cortex (including piriform cortex)	[159,193]	cortex (including entorhinal cortex)	[194,195,196,197,198]
	caudate-putamen	[159,193,199]	hippocampus	[194,195,196,197,198,200]
	nucleus accumbens	[159,193]	caudate-putamen	[194,195,196,197,198,201]
	hippocampus	[159,193,202,203]	nucleus accumbens	[196,197,198]
	thalamus	[159]	subiculum	[196,197]
	hypothalamus	[159]	thalamus	[196,198]
	subiculum	[159]	hypothalamus	[196]
	cerebellum	[159]	inferior and superior colliculi	[196,198]
	inferior colliculus	[193]	amygdala	[200]
	olfactory bulb	[159,193]	olfactory bulb	[196,198]
	olfactory tubercule	[159,193]	olfactory tubercule	[196,197]
**mGlu_2_**	cortex (including piriform cortex, entorhinal cortex)	[114,204,205]		
	hippocampus	[114,202,204,205]	cortex (including piriform cortex, entorhinal cortex)	[194,197,206,207]
	thalamus	[114,204,205]	hippocampus	[194,197,207]
	basolateral amygdala	[114,204]	thalamus	[197,206,207]
	caudate-putamen	[114,204,205]	basolateral amygdala	[206,207]
	nucleus accumbens	[114,204]	caudate-putamen	[206]
	globus pallidus	[204]	nucleus accumbens	[206]
	substantia nigra	[204]	globus pallidus	[206]
	ventral tegmental area	[114]	cerebellum	[194,197,206,208]
	cerebellum	[114,204]	olfactory tubercule	[206]
	olfactory bulb	[114,204]		
	olfactory tubercule	[114,204]		
**mGlu_4_**	cortex (including piriform cortex)	[209]	cortex (including entorhinal cortex)	[194,197,210,211,212]
	caudate-putamen	[209]	caudate-putamen	[197,210,212,213]
	substantia nigra	[209]	substantia nigra	[197]
	hippocampus	[202,209]	nucleus accumbens	[197,212,213]
	thalamus	[209]	thalamus	[194,197,210,212,213,214]
	hypothalamus	[209]	hypothalamus	[212]
	amygdala	[209]	hippocampus	[194,210,214]
	superior colliculus	[209]	amygdala	[212]
	cerebellum	[209,215,216]	lateral septum	[210,214]
	olfactory bulb	[209]	cerebellum	[194,197,208,212,214]
	olfactory tubercule	[209]	olfactory bulb	[210,212,214]
			olfactory tubercule	[197,214]
**mGlu_7_**	cortex (including piriform cortex)	[136,217]		
	caudate-putamen	[136]		
	nucleus accumbens	[136]		
	globus pallidus	[136]		
	substantia nigra	[136]		
	thalamus	[136]		
	hypothalamus	[136]		
	hippocampus	[136]		
	subiculum	[136]		
	amygdala	[136]	cortex	[212,218,219,220]
	ventral tegmental area	[136]	caudate-putamen	[212,213,218,219,220]
	olfactory bulb	[136]	globus pallidus	[212]
	olfactory tubercule	[217]	nucleus accumbens	[212,213,218,220]
			substantia nigra	[212]
	(mGlu_7a_)		thalamus	[212,213,218,219,220]
	
	cortex	[136]	hypothalamus	[212,219,220]
	hippocampus	[136]	amygdala	[212,220]
	substantia nigra	[136]	hippocampus	[218,219,220]
	globus pallidus	[136]	ventral tegmental area	[212]
	amygdala	[136]	superior and inferior colliculi	[219]
	cerebellum	[136]	locus coeruleus	[218]
	
	(mGlu_7b_)		cerebellum	[208,212,218,219,220]
	
	cortex (including piriform cortex)	[221,222]	olfactory bulb	[212,218,219]
	hippocampus	[202,221,222,223]	olfactory tubercule	[219,220]
	thalamus	[222]		
	caudate-putamen	[222]		
	globus pallidus	[222]		
	nucleus accumbens	[222]		
	locus coeruleus	[222]		
	cerebellum	[222]		
	olfactory bulb	[221]		
**mGlu_8_**			cortex (including piriform cortex)	[218,224,225]
			striatum	[213,218,225]
			nucleus accumbens	[213,225]
			globus pallidus	[225]
	piriform cortex	[216]	substantia nigra	[225]
	entorhinal cortex	[216]	thalamus	[213,218,224,225]
	hippocampus	[202,226]	hypothalamus	[225]
	olfactory bulb	[216]	hippocampus	[218,224,225,226]
			amygdala	[218,225]
			cerebellum	[208,218,224,225]
			olfactory bulb	[218,224,227]
			olfactory tubercule	[227]
**(B)**				
**Receptor**	**Protein**		**mRNA**	
**M_1_**	frontal cortex	[69,79,227]		
	parietal cortex	[70,228]		
	temporal cortex	[228]	frontal cortex	[70]
	occipital cortex	[228]	parietal cortex	[70]
	primary visual cortex	[80]	thalamus	[79]
	thalamus	[79,80]	hippocampus	[75]
	hippocampus	[80,228]	caudate-putamen	[77]
	nucleus basalis	[228]		
	putamen	[228]		
**M_4_**	frontal cortex	[70,228]		
	temporal cortex	[228]		
	parietal cortex	[70,228]	frontal cortex	[70]
	occipital cortex	[228]	parietal cortex	[70]
	thalamus	[79]	thalamus	[79]
	hippocampus	[228]	hippocampus	[75]
	nucleus basalis	[228]		
	putamen	[228]		
**M_5_**	frontal cortex	[228]		
	temporal cortex	[228]		
	parietal cortex	[228]		
	occipital cortex	[228]		
	nucleus basalis	[228]		
**GABA_B_**			prefrontal cortex	[229]
			frontal cortex	[192]
			occipital cortex	[192]
			temporal cortex	[192]
			caudate nucleus	[192,229]
			putamen	[192,229]
			globus pallidus	[229]
			substantia nigra	[192,229]
			nucleus accumbens	[192]
	entorhinal cortex	[230]	thalamus	[192]
	caudate	[230]	hypothalamus	[192]
	putamen	[230]	hippocampus	[192,229]
	globus pallidus	[230]	amygdala	[192]
	thalamus	[230]	corpus callosum	[192]
	hippocampus	[230]	cerebellum	[192,229]
	substantia nigra	[230]		
	cerebellum	[230]		
			cortex	[191]
	(GABA_B1_, GABA_B2_)		putamen	[191]
			caudate nucleus	[191]
			substantia nigra	[191]
			thalamus	[191]
			hippocampus	[191]
			amygdala	[191]
			cerebellum	[191]
			(GABA_B2_)	
**mGlu_5_**	frontal cortex	[94]	cortex (including frontal cortex, prefrontal cortex)	[89,94,153]
	hippocampus	[231]	hippocampus	[90,153]
	lateral cerebellum	[94]	parahippocampal gyrus	[90]
			cerebellum	[94,153]
**mGlu_2_**	prefrontal cortex	[103]	prefrontal cortex	[102]
	temporal cortex	[103]	thalamus	[91]
	dorsolateral prefrontal cortex	[100]	hippocampus	[101]
	motor cortex	[103]	ventral mesencephalon (including substantia nigra)	[102]
	hippocampus	[231]		
**mGlu_4_**	hippocampus	[231]	cortex	[232]
			putamen	[232]
			substantia nigra	[232]
			caudate nucleus	[232]
			thalamus	[91,232,233]
			hypothalamus	[232,233]
			hippocampus	[232,233]
			amygdala	[232]
			corpus callosum	[232]
			cerebellum	[232,233,234]
**mGlu_7_**			cortex (including entorhinal cortex)	[235]
			thalamus	[91,234,235]
			hypothalamus	[234]
			hippocampus	[234,235]
			caudate-putamen	[235]
			cerebellum	[235]
**mGlu_8_**			cortex	[133]
			putamen	[133,225]
			caudate nucleus	[133,225]
			globus pallidus	[225]
			nucleus accumbens	[225]
			substantia nigra	[225]
			cingulate gyrus	[225]
			thalamus	[91,133,225]
			hypothalamus	[225]
			hippocampus	[225]
			amygdala	[133,225]
			locus coeruleus	[225]
			cerebellum	[133,225]

The quantitative analysis of the expression of the receptors differed between structures and comparisons, which may modulate the development of therapeutic effects and adverse effects. The quantitative analyses of the receptors in the brain structures most important for schizophrenia pathophysiology and treatment are summarized in Table 5.

Schematics of the rat and human brains with the expression of receptor proteins in outlined areas are also provided (as shown in Figure 2, Figure 3, Figure 4, Figure 5, Figure 6, Figure 7 and Figure 8), where the differences in the intensity of the expression of receptors are schematically visualized. These figures were constructed to show differences in the expression of individual receptors in different structures.

Comparisons of the intensity of receptor expression with the antipsychotic efficacy of ligands activating these receptors clearly show that the activity of the ligands does not necessarily correspond with the intensity of receptor expression in relevant structures. Therefore, orthosteric agonists or PAMs of mGlu_4_ receptors exhibit excellent activity in animal models of schizophrenia [130,236], but these receptors are expressed at the lowest levels in the cortex and hippocampus compared to other brain areas [209,232]. Instead, the high expression of mGlu_4_ receptors in the globus pallidus, where it is a heteroreceptor on GABAergic terminals, makes it a good target for anti-Parkinson drugs [237]. However, stimulation of these receptors may increase the risk of adverse effects on non-Parkinson patients. Much lower doses of mGlu_4_ PAMs/orthosteric agonists were active in animal models of schizophrenia than in models of Parkinson’s disease [237]. Therefore, the risk of inducing adverse effects during antipsychotic treatment appears to be relatively low.

The extensive expression of GABA_B_ and mGlu_5_ receptors in cortical structures and the hippocampal formation [187,190] and their lower expression in deeper brain structures positively correlate with the activity of their ligands in animal models of schizophrenia and exclusively support the use of these receptors as targets for antipsychotic drugs. The functional connection of mGlu_5_ with NMDA receptors increases the risk of inducing adverse effects with activation of mGlu_5_ receptors, but biased ligands may be a solution [53].

Despite the initial hopes for mGlu_2_ receptors as antipsychotic drug targets, their expression in the cortex and hippocampus is relatively low [204].

The high expression of muscarinic receptors in structures related to schizophrenia arousal makes them excellent antipsychotic drug targets [163,238], and the efficacy of compounds activating these receptors was confirmed in animal models [152,169]. Of the three analyzed receptors, M_1_ was expressed at the highest levels.

**Figure 9:**
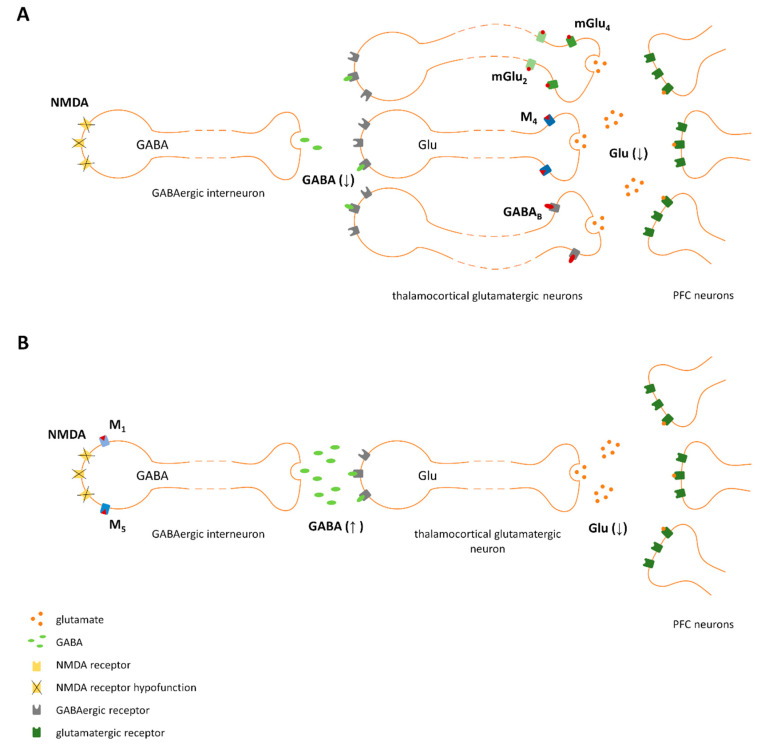
Proposed mechanism of action of ligands activating pre- (**A**) and postsynaptic receptors (**B**). NMDA receptor hypofunction results in decreased GABA release from GABAergic interneurons, which leads to disinhibition of thalamocortical glutamatergic neurons and increased glutamate release in the prefrontal cortex (PFC). A reduction in excess glutamate release in the PFC could be achieved directly (**A**) by the activation of presynaptic receptors expressed on thalamocortical glutamatergic terminals. (e.g., mGlu_2_, mGlu_4_, M_4_, or GABA_B_) or indirectly (**B**) by stimulating GABA release via the activation of postsynaptic receptors expressed on GABAergic interneurons (e.g., mGlu_5_, M_1_, or M_5_).

The direct stimulation of post- or presynaptic sites results in the regulation of a particular neuron, which subsequently affects the neurons it innervates. The mechanisms engaged in the stabilization of inhibitory-excitatory balance in the CNS that are responsible for the antipsychotic effects of compounds are schematically shown in Figure 9.

The aim of successive psychotropic treatment is to maintain homeostatic balance in the brain. Due to the extraordinary complexity of the central nervous system and its sensitivity to external factors, the precision and sensitivity of pharmacological manipulations must be considered to avoid adverse effects due to the unnecessary effects on the neuronal pathways responsible for other brain activities and functions.

## 4. Strategies Based on Bidirectional Inhibition of Glutamate Release

The individual differences between subjects, the complexity of microcircuits that regulate basic processes and the expression of receptors within these microcircuits have not been fully recognized in patients with schizophrenia and may determine the effectiveness and safety of treatment. Although several studies and clinical trials have been conducted, the treatment of negative and cognitive symptoms of schizophrenia remains unsatisfactory. Extensive research has been performed to develop new solutions, but spectacular success is lacking.

Exclusive stimulation of the receptors expressed in neuronal circuits involved in the pathophysiology of schizophrenia, without effects on dopaminergic neurotransmission and/or NMDA receptor-mediated signaling, should minimize the risk of adverse effects and improve the effectiveness of therapy. Our recent studies proposed a treatment based on the simultaneous stimulation of two receptors that are crucial for regulation of glutamatergic networks, and the results have been published [138,152,169,170,236,239]. In these studies, select combinations activating mGlu_2_/M_1_, mGlu_2_/M_5_, and mGlu_4_/M_4_ were not shown to alter prolactin levels or locomotor activity [152,170], prompting us to speculate that the use of sub-effective doses of at least two ligands may be safer than the highest dose of each compound alone or in combination with D_2_-based drugs [169,170].

The studies were performed using ligands that activate the receptors described in the first part of this review, e.g., muscarinic M_1_, M_4_ and M_5_, GABA_B_ and metabotropic glutamate receptors (mGlu_2_, mGlu_4_ and mGlu_5_ receptors). Different combinations of ligands were used, and their efficacies were investigated by performing a vast range of tests in rodents that reflected the positive, negative, and cognitive symptoms of schizophrenia (Table 6).

### 4.1. Simultaneous Administration of Ligands Activating Receptors Associated with Adenyl Cyclase Activity

The investigated combinations of ligands and their efficacies in animal models are shown in Table 7. The best working pair of compounds with evident efficacy in models of the positive, negative, and cognitive symptoms of schizophrenia were ligands that activated mGlu_4_/M_4_ receptors and mGlu_2_/M_4_ receptors (although these drugs were not tested in the models of positive symptoms) [152,239]. The simultaneous activation of GABA_B_ receptors with mGlu_4_ or M_4_ receptors was not effective in models of negative symptoms and/or cognitive decline [169,236], and thus these combinations are less attractive for the reversal of negative and cognitive symptoms. However, the simultaneous activation of GABA_B_/M_4_ or mGlu_4_ receptors may be safer and more effective in patients with positive symptoms because the treatment of positive symptoms using current neuroleptic drugs carries a high risk of adverse effects.

The synergistic effects of ligands with affinity for two different presynaptically located receptors may result from several factors:

The receptors are localized on one axon terminal, putatively a glutamatergic terminal. The concomitant stimulation results in the inhibition of glutamate release, and the ligands may complement the action of the other ligand. The receptors may act separately or through heterodimer formation (for a detailed description, see Section 4.1.1)

The receptors are localized on different nerve endings that innervate one brain area and/or several different structures. The receptors may complement the action of the other in that area, as shown in Figure 9.

#### 4.1.1. Heterodimerization

As mentioned above, G protein-coupled receptors are known to form homo- and heteromeric structures. In the physiological state, mGlu receptors function as homodimers composed of two identical subunits, and each subunit may both bind the ligand and activate G-protein signaling (for a review see: Wieronska et al., 2016 [51]). The GABA_B_ receptor functions as a heterodimer composed of two subunits, GABA_B1_ and GABA_B2_. The subunits depend on each other, i.e., GABA_B1_ binds the ligand and GABA_B2_ activates the signal transduction pathway [240].

According to numerous reports, G protein-coupled receptors may form heterodimers or oligomers with the same or other types of receptors, indicating strong multiple interactions between two or more receptors [241,242,243,244,245,246,247]. mGlu_2_-5-HT_2A_ heterodimerization is one of the most important pathways implicated in schizophrenia [248,249,250]. Other forms of heterocomplexes in relation to schizophrenia have also been described, such as the mGlu_5_/D_2_/A_2A_ oligomer [251,252]. Recently, mGlu_2_/mGlu_4_ heterodimers were described [253,254,255,256]. Therefore, the possible heterodimeric or oligomeric interactions of mGlu and muscarinic receptors are open for investigation and may possibly be implicated in the pathophysiology and treatment of schizophrenia.

### 4.2. Simultaneous Administration of Ligands Activating Receptors Associated with Adenyl Cyclase and the Inositol Phosphate Signaling Pathway

As shown in Table 8, the activity of the combined administration of sub-effective doses of an allosteric agonist of M_1_ or PAM of M_5_ receptors with sub-effective doses of PAMs of mGlu_2_ or GABA_B_ receptors was observed in models of the cognitive symptoms of schizophrenia, but not in the models of positive symptoms [170]. No activity of the allosteric ligands of M_1_ or M_5_ receptors was observed in models of negative symptoms of schizophrenia [170]. Therefore, their combinations with ligands activating mGlu_2_ or GABA_B_ receptors were not tested.

The costimulation of GABA_B_-mGlu_5_ receptors exhibited clear and evident efficacy in models of the positive, negative and cognitive symptoms of schizophrenia, which were comparable to the effects of the active dose of each ligand administered alone [138].

The expression of the receptors supports different mechanisms of the synergistic effects than the presynaptically expressed receptors.

Most likely, the postsynaptic receptors mGlu_5_, M_1,_ and M_5_ are expressed on GABAergic neuron somata and dendrites [147,257,258,259], which enhances GABAergic inhibitory currents, and this activation indirectly counteracts GABAergic dysfunction due to NMDA hypofunction.

As indicated above, the activation of mGlu_2_ or GABA_B_ receptors inhibits glutamate release. Therefore, the dual action involves an increase in the inhibition on the one hand and the inhibition of excitation on the other hand, which restores brain homeostasis.

## 5. Conclusions

**Figure 10:**
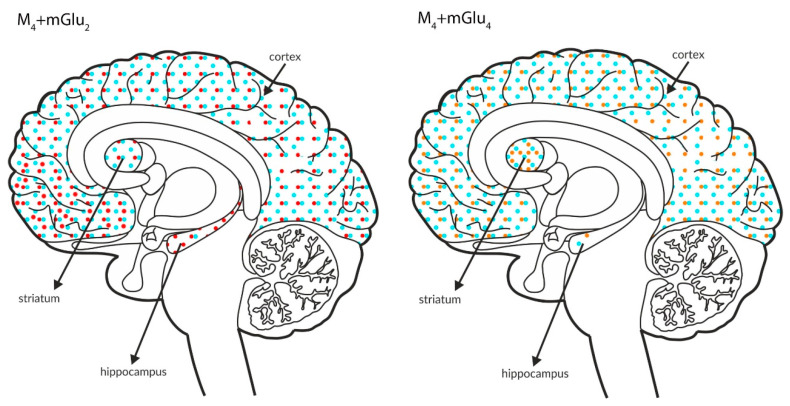

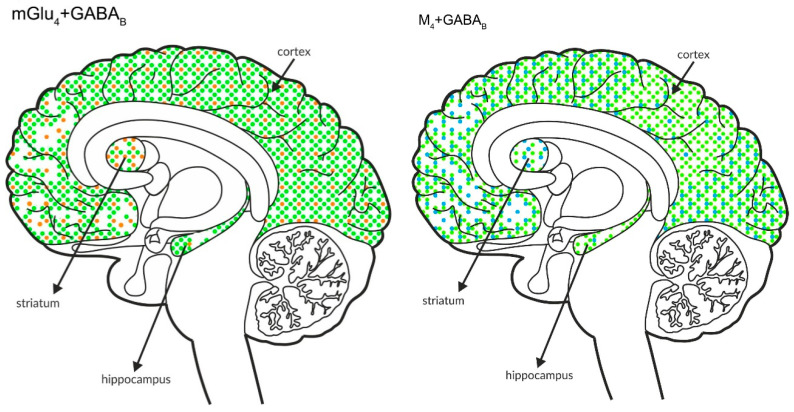
Simultaneous presynaptic effects on glutamate release. The coexpression of M_4_ receptors with mGlu_2_, GABA_B_ or mGlu_4_ and mGlu_4_ with GABA_B_ receptors in the cortex, hippocampus, and striatum of the human brain. M_4_ receptors are shown in light blue (_•_), mGlu_2_ is shown in red (_•_), mGlu_4_ is shown in orange (_•_), and GABA_B_ is shown in neon green (_•_).

**Figure 11:**
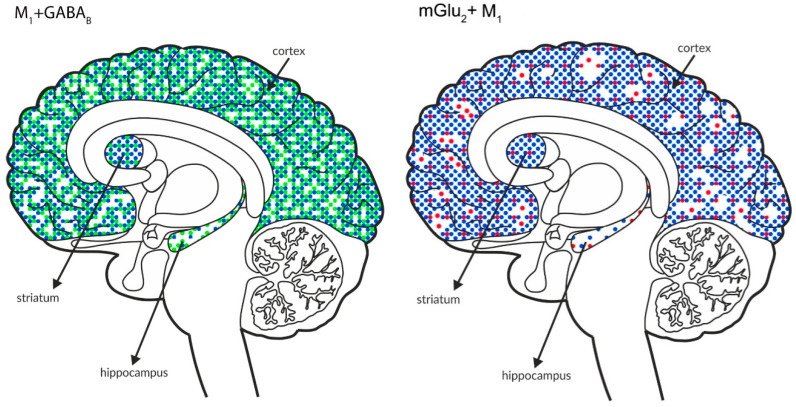

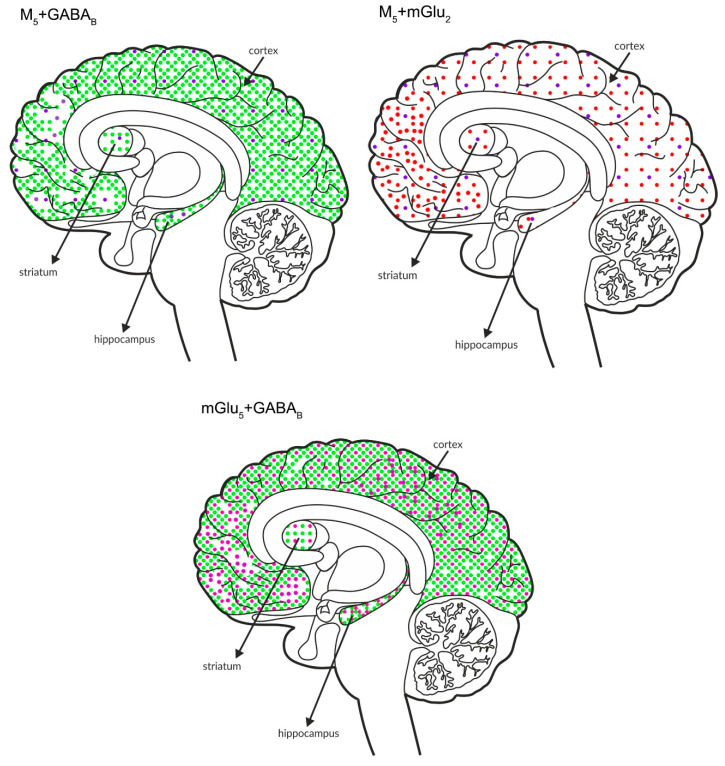
Simultaneous pre- and postsynaptic effects on glutamate release. The coexpression of M_1_ receptors with GABA_B_ and mGlu_2_, M5 receptors with GABA_B_ and mGlu_2_ receptors and mGlu_5_ receptors with GABA_B_ receptors in the cortex, hippocampus, and striatum of the human brain. M_1_ receptors are shown in navy blue (_•_), M_5_ receptors are shown in violet (_•_), mGlu_2_ is shown in red (_•_), GABA_B_ is shown in neon green (_•_), and mGlu_5_ is shown in neon pink (_•_).

The figures shown below (Figure 10 and Figure 11) schematically illustrate the coexistence of particular types of receptors in select structures.

The benefits and advantages of the combined activation of two selected receptors are sufficient to support the use of this approach in the treatment of schizophrenia.

Neither of the proposed treatments are based on the inhibition of dopaminergic receptors. Therefore, it may be speculated that the treatments are less burdened with the induction of adverse effects such as motor coordination and prolactin levels that are typical for presently used typical and second-generation neuroleptics. Preliminary experimental results supporting such conclusions can be found in Cieslik et al. 2018, Cieslik et al. 2019, and Cieslik et al. 2020 [152,169,170].

The results presented in the studies by Cieslik et al. 2018; 2020 indicate that the combined administration of the highest doses of the compounds or the administration of the highest dose of one compound with a subactive dose of the other does not produce additive effects [152,170]. Thus, the dosage does not need to be increased, and subsequently, the risk of unnecessary exposure to a treatment to obtain a therapeutic effect is relatively low. This finding might indicate the limited risk of unexpected events or toxic effects due to the accidental administration of a double dose of medications, which is particularly important for the mGlu_4_ or GABA_B_ receptor_._ As stated above, the mGlu_4_ receptor, which is expressed in striatopallidal pathways, is considered an antiparkinsonian target [46,260]. The overstimulation of the receptor in these brain areas may result in undesired effects that counteract the putative antipsychotic efficacy. On the other hand, overstimulation of the GABA_B_ receptor may exert adverse effects, such as sedation [139,140,261].

Analyses of the figures show overlap in the expression of particular receptors in select brain areas. The activation of receptors that are expressed at lower levels, such as mGlu_2_ or mGlu_4,_ together with other types of receptors that are expressed at higher levels may complement the efficacy of the other receptor.

Overall conclusions obtained from the results discussed above and the consequences of the simultaneous administration of two compounds are as follows:

− the dose of each compound may be reduced and the antipsychotic-like efficacy is the same as the highest dose of each compound administered alone (this approach may potentially allow us to avoid putative adverse effects or unnecessary exposure of the prodrug to patients, as shown previously for mGlu_2/3_ agonists);
− the action of the combined treatment might be selective in specific areas and thus may target a specific group of symptoms;
− the ligands administered in combinations may complement the action of the other ligand and compensate for possible receptor dysfunctions, activating both homodimers and heterodimers/heterocomplexes.

## Figures and Tables

**Table 1:** Groups of symptoms and symptom intensity in patients with schizophrenia, schizoaffective 31disorder, and psychotic disorder, where “−”no symptoms, “+”—very mild, “++”—mild, “+++”—moderate, “++++”—severe,” +++++”—very severe (based on [3]).

Schizophrenic Subtype/Disorder	Positive Symptoms	Negative Symptoms	Cognitive Symptoms	Psychomotor Disturbances
paranoid	+++++	−	−	−
disorganized	−	++	+++++	−
catatonic	−	++	++	+++++
unspecified	+++	+++	+++	++
residual	−	++++	+	
Schizoaffective disorder	+++	+++	−	−
brief psychotic disorder	++	+++++	−	++

**Table 2:** Select reviews describing the role of metabotropic glutamate receptors in schizophrenia.

Chaki et al., 2010	[35]	mGlu_2/3_
Lesage et al., 2010	[36]	mGlu_1_
Marek, 2010	[37]	mGlu_2/3_
Yasuhara et al., 2010	[38]	mGlu_1_, mGlu_2_, mGlu_2/3_, mGlu_5_
Chaki et al., 2011	[39]	mGlu_1_, mGlu_2_, mGlu_2/3_
Gregory et al., 2011	[40]	mGlu_2_, mGlu_5_
Nicoletti et al., 2011	[41]	mGlu_1_, mGlu_2/3_, mGlu_5_
Sheffler et al., 2011	[42]	mGlu_2_, mGlu_5_
Fell et al., 2012	[43]	mGlu_2_, mGlu_2/3_
Vinson et al., 2012	[44]	mGlu_2_, mGlu_3_, mGlu_5_
Gregory et al., 2013	[45]	mGlu_2/3_, mGlu_5_
Nickols et al., 2014	[46]	mGlu_2_, mGlu_2/3_, mGlu_4_, mGlu_5_
Li et al., 2015	[47]	mGlu_2/3_
Golubeva et al., 2016	[48]	mGlu_2_, mGlu_2/3_, mGlu_4_, mGlu_5_, mGlu_7_
Walker et al., 2015	[49]	mGlu_1_, mGlu_2_, mGlu_3_, mGlu_2/3_, mGlu_5_
Muguruza et al., 2016	[50]	mGlu_2/3_
Wierońska et al., 2016	[51]	mGlu_2/3_, mGlu_5_, mGlu_4_, mGlu_7_
Foster et al., 2017	[52]	mGlu_1_, mGlu_2_, mGlu_3_, mGlu_2/3_, mGlu_5_
Maksymetz et al., 2017	[53]	mGlu_1_, mGlu_2_, mGlu_3_, mGlu_4_, mGlu_5_, mGlu_7_, mGlu_8_
Nicoletti et al., 2019	[54]	mGlu_1_, mGlu_2_, mGlu_2/3_, mGlu_4_, mGlu_5_
Stansley et al., 2019	[55]	mGlu_1_, mGlu_3_

**Table 5:** Comparison of the expression of muscarinic (M_1_, M_4_, and M_5_), GABA_B_ and metabotropic glutamate (mGlu_2_, mGlu_4_, and mGlu_5_) receptors in select brain structures: “0”—not detected, “+”—very low, “++”—low, “+++”—moderate, “++++”—high,” +++++”—intense, “nd”—no data.

	M_1_	M_4_	M_5_	GABA_B_	mGlu_2_	mGlu_4_	mGlu_5_
cortex	+++++	+++	+	++++	++	++	++/+++
hippocampus	+++	nd	+	+++++	+++	+	++++
striatum	+++++	+++	+	+++/++++	++	+++	++
hypothalamus	nd	nd	nd	++++	0/+	+++	+
thalamus	++	+++	+	++++	++	+++	++
amygdala	nd	nd	nd	+++	+/++	+++	+/++
cerebellum	nd	nd	nd	++++	+++	+++++	++

**Table 6:** Tests used to assess the antipsychotic activity of investigated ligands in rodents.

Positive Symptoms	Negative Symptoms	Cognitive Symptoms
DOI-induced head twitches Amphetamine-induced hyperlocomotion MK-801-induced hyperlocomotion	Social interactions Modified forced swim test	Novel object recognition Spatial alterations Prepulse inhibition

**Table 7:** Efficacy of the investigated combinations of ligands in tests assessing antipsychotic activity in rodents: “+”—compounds reversed the induced disruptions, “−/+”—compounds showed a trend toward reversing the induced disruptions, and “−”—compounds had no effect on the induced disruptions.

Synaptic Localization		Behavioral Test	Activity
Pre	Pre		
**mGlu_2_**	**M_4_**	social interaction test	+
		novel object recognition test	+
**mGlu_4_**	**M_4_**	DOI-induced head twitches	−/+
		MK-801-induced hyperactivity	+
		AMPH-induced hyperactivity	+
		modified forced swim test	+
		social interaction test	+
		novel object recognition test	+
**GABA_B_**	**mGlu_4_**	DOI-induced head twitches	+
		MK-801-induced hyperactivity	+
		social interaction test	−
		novel object recognition test	−
**GABA_B_**	**M_4_**	DOI-induced head twitches	+
		social interaction test	−
		novel object recognition test	+

**Table 8:** Efficacy of investigated combinations of ligands in tests assessing antipsychotic activity in rodents: “+”—compounds reversed the induced disruptions and “−”—compounds had no effect on the induced disruptions.

Synaptic Localization		Behavioral Test	Activity
Pre	Post		
**mGlu_2_**	**M_1_**	novel object recognition test	+
		prepulse inhibition	+
		spatial-delayed alternation test	+
**mGlu_2_**	**M_5_**	novel object recognition test	+
		prepulse inhibition	+
		spatial-delayed alternation test	+
**GABA_B_**	**mGlu_5_**	modified forced swim test	+
		social interaction test	+
		novel object recognition test	+
**GABA_B_**	**M_1_**	DOI-induced head twitches	−
		novel object recognition test	+
**GABA_B_**	**M_5_**	DOI-induced head twitches	−
		novel object recognition test	+
